# Primary Ewing’s Sarcoma of the Kidney Mimicking Renal Cell Carcinoma With Widespread Metastasis: A Case Report and a Brief Review of the Literature

**DOI:** 10.7759/cureus.64896

**Published:** 2024-07-19

**Authors:** Varsha Rangankar, Aryaman Dhande, Sanjay M Khaladkar, Prajakta P Kirdat Patil, Gayatri Bhuibhar

**Affiliations:** 1 Radiodiagnosis, Dr. D. Y. Patil Medical College, Hospital and Research Centre, Dr. D. Y. Patil Vidyapeeth, Pune, Pune, IND; 2 Pathology, Dr. D. Y. Patil Medical College, Hospital and Research Centre, Dr. D. Y. Patil Vidyapeeth, Pune, Pune, IND

**Keywords:** urology, metastasis, thrombosis, kidney, extraosseous ewing’s sarcoma, renal cell carcinoma

## Abstract

Ewing's sarcoma family of tumors (ESFTs) are a group of small round cell tumors with common morphological and genetic features, including Ewing's sarcoma of bone, primary extra-skeletal Ewing tumors, extraosseous Ewing sarcoma (EES), and Askin tumors. EES presenting as a primary renal mass is an exceedingly uncommon aggressive tumor with limited reported cases in the literature and often mimics other renal malignancies on imaging. We present a case of a 31-year-old man presenting with left flank pain and abdominal fullness of short duration. Radiological imaging studies showed a large heterogenous mass from the left kidney, confirmed to be Ewing’s sarcoma on post-operative histopathological examination (HPE) and immunohistochemistry (IHC) studies. Subsequent follow-up showed extensive metastatic disease. EES of the kidney has a nonspecific presentation and imaging appearance necessitating a multi-disciplinary approach comprising radiological imaging with a high index of suspicion, HPE, IHC, and molecular analysis for the correct diagnosis.

## Introduction

Ewing's sarcoma family of tumors (ESFTs) encompass a group of genetically related high-grade round cell tumors, originating from common mesenchymal stem cells [1,2]. ESFT includes Ewing’s sarcoma of bone (ESB), primary extra-skeletal Ewing tumors, also known as peripheral primitive neuroectodermal tumors (PNET), extraosseous Ewing sarcoma (EES), and Askin tumors. ESFT stands out as one of the rarest neoplastic conditions, warranting heightened attention from healthcare providers. Due to its atypical presentation, ESFT often presents challenges in initial diagnosis and management compared to more commonly encountered sarcomas [3-6]. EES, first described by Angervall and Enzinger in 1975, was initially considered a separate entity from other ESFT tumors and later proved to have common histological and genetic features [6,7].

EES most commonly occurs at primary sites which include intracranial locations, paravertebral regions, thoraco-pulmonary areas (Askin tumors), extremities, kidneys, adrenal glands, pancreas, gastrointestinal tract, retroperitoneum, ovaries, and urinary bladder [2]. EES presenting as a primary renal mass is an exceedingly uncommon occurrence, with a reported incidence of slightly over 100 cases worldwide [1,8]. Among the primary malignancies of the kidney, renal PNET/EES accounts for only 1%, with the majority of primary renal tumors being renal cell carcinoma (RCC) [9]. Nonetheless, accurately diagnosing PNET/EES of the kidney is crucial, as it represents a notably more aggressive renal tumor affecting relatively younger adults. Clinically, EES of the kidney commonly manifests with nonspecific symptoms such as flank pain, haematuria, and palpable abdominal masses. Given its rarity and similarity to other renal neoplasms, achieving an accurate diagnosis can be challenging, necessitating a multidisciplinary approach involving radiological imaging, histopathological examination (HPE), and molecular analysis [9].

A 31-year-old male patient presented with a large, solid renal mass lesion that was initially diagnosed as primary RCC based on cross-sectional imaging findings. However, HPE revealed that the patient had an EES kidney. We present this case report highlighting clinical, imaging, and histopathological findings with subsequent follow-up of the metastatic disease.

## Case presentation

A 31-year-old male patient was referred to our tertiary care hospital with left flank pain and abdominal fullness for 12 days duration and frank hematuria for seven days duration. The patient had no other symptoms, operative history, or comorbidities. On clinical examination, a large palpable mass was present in the left hypochondrium and left lumbar region. He had reduced hemoglobin (9.80 gm/dL), red blood cell count (3.24 million cells/µL), and hematocrit (20%) levels. Other blood investigations, including renal and liver function tests and coagulation profiles, were within normal range.

**Figure 1:**
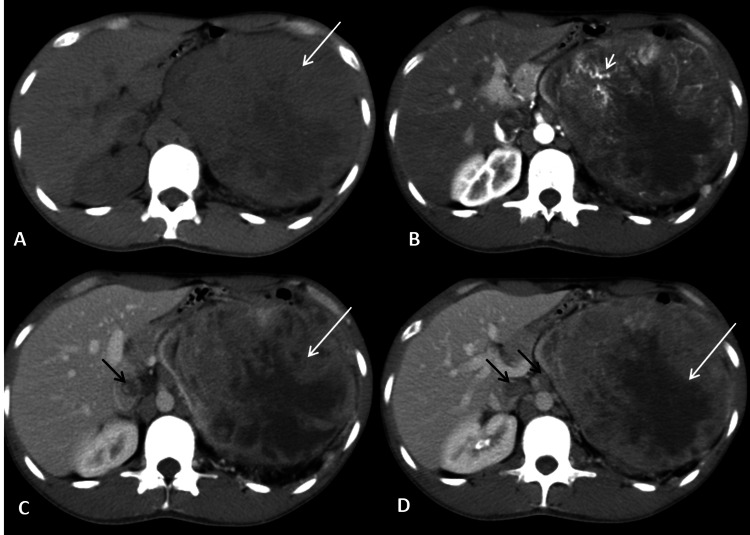
Axial NCCT image revealed a large heterogeneous mass in the left renal fossa (A, long arrow), showing neovascularization on arterial phase images (B, short arrow). The mass demonstrated heterogeneous post-contrast enhancement with non-enhancing necrotic areas within on the venous phase (C, D, long arrow). Heterogeneous enhancing tumor thrombosis was seen in the IVC and left renal vein (C, D, black arrows). NCCT: non-contrast computed tomography; IVC: inferior vena cava

Contrast-enhanced computed tomography (CECT) study of the abdomen (Figures 1A-1D) revealed a large heterogeneously enhancing mass lesion involving the anterior cortex of the left kidney showing neovascularity with tumor thrombus in the left renal vein and inferior vena cava (IVC). A few lytic lesions were also noted in the inferior pubic ramus and left 10th rib which was suspicious for metastasis.

**Figure 2:**
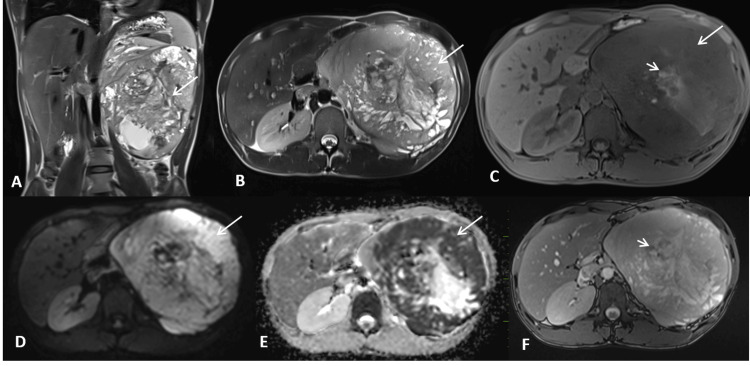
T2-HASTE coronal and axial images reveal a large heterogeneously hyperintense mass lesion in the left renal fossa with cystic areas (A, B; long arrow). The mass shows significant diffusion restriction (D, long arrow) with corresponding low apparent diffusion coefficient (ADC) values (E, long arrow). A few hyperintense areas on Dixon fat-suppressed images (C, short arrow) exhibit blooming on TRUFI (F, short arrow), indicating hemorrhage. HASTE: half-Fourier acquisition single-shot turbo spin-echo; TRUFI: true fast imaging with steady-state free precession

**Figure 3:**
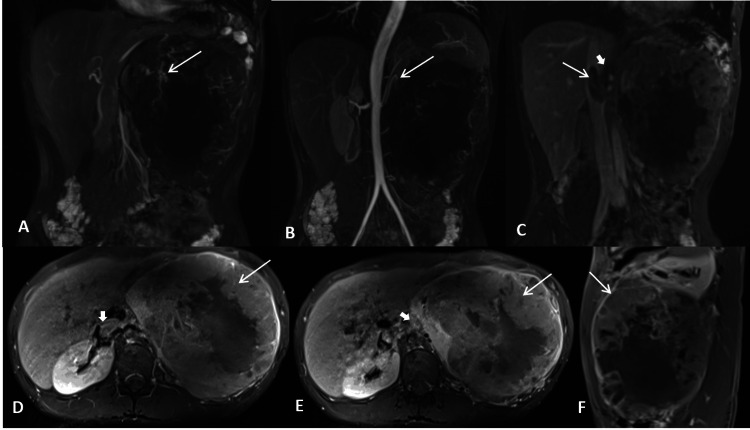
Coronal reformatted MIP MR angiography images reveal a mass in the left renal fossa showing marked neovascularity (A, long arrow) and supplied by the left renal artery (B, long arrow). Coronal reformatted MIP venography images demonstrate a hypointense thrombus in the inferior vena cava (IVC) (C, long arrow) and left renal vein (C, short arrow). Axial post-contrast T1FS images show heterogeneous post-contrast enhancement of the mass (D, E, long arrow) and heterogeneously enhancing tumor thrombus in the IVC and left renal vein (D, E, short arrow). A thick enhancing capsule is noted at the periphery of the lesion on the sagittal post-contrast T1FS image (F, long arrow). MIP: maximum intensity projection; FS: fat-saturated

Contrast-enhanced magnetic resonance imaging (CE-MRI) of the abdomen with angiogram revealed a large, predominantly solid mass lesion arising from the left kidney's interpolar and lower pole region, predominantly involving the lateral and anterior cortex (Figures 2A-2F). The lesion measured 18 x 14.5 x 14.1 cm in craniocaudal (CC), anteroposterior (AP), and transverse (TR) dimensions respectively. The lesion showed marked diffusion restriction and areas of hemorrhage. Neovascularity and heterogeneous enhancement were evident in post-contrast sequences with enhancing tumor thrombus in the left renal and IVC (Figures 3A-3F). An enhancing capsule was also seen around the mass lesion on delayed post-contrast images (Figure 3F). The lesion was invading the Gerota's fascia and causing a significant mass effect on the adjacent structures with dilatation of the pelvicalyceal system in the upper pole of the left kidney. Based on imaging findings, a diagnosis of left renal neoplasm, likely RCC (T3b/T4 by American Joint Committee on Cancer (AJCC TNM) staging system), was proposed. Other possibilities included mesenchymal tumors such as renal sarcomas.

**Figure 4:**
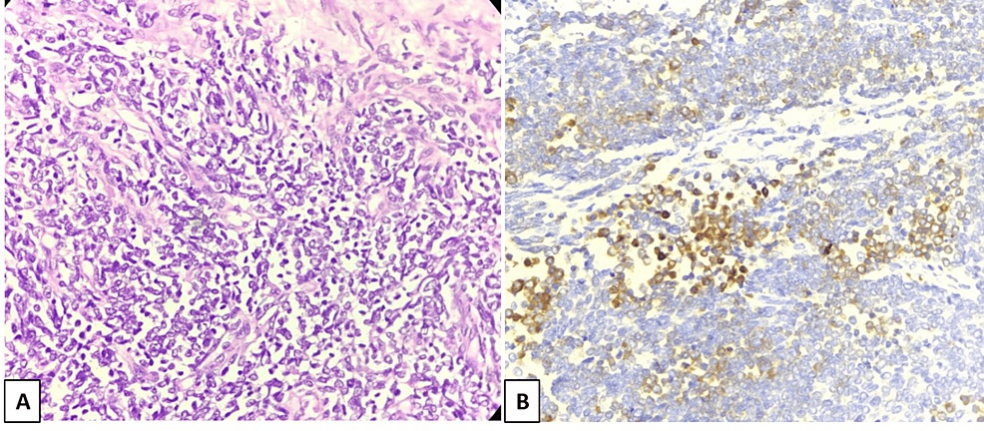
Histopathological examination (HPE) shows small ovoid tumor cells with irregular hyperchromatic nuclei and scant cytoplasm (A, H&E, 400X). Immunohistochemical (IHC) staining reveals synaptophysin positivity (B, 400X).

**Figure 5:**
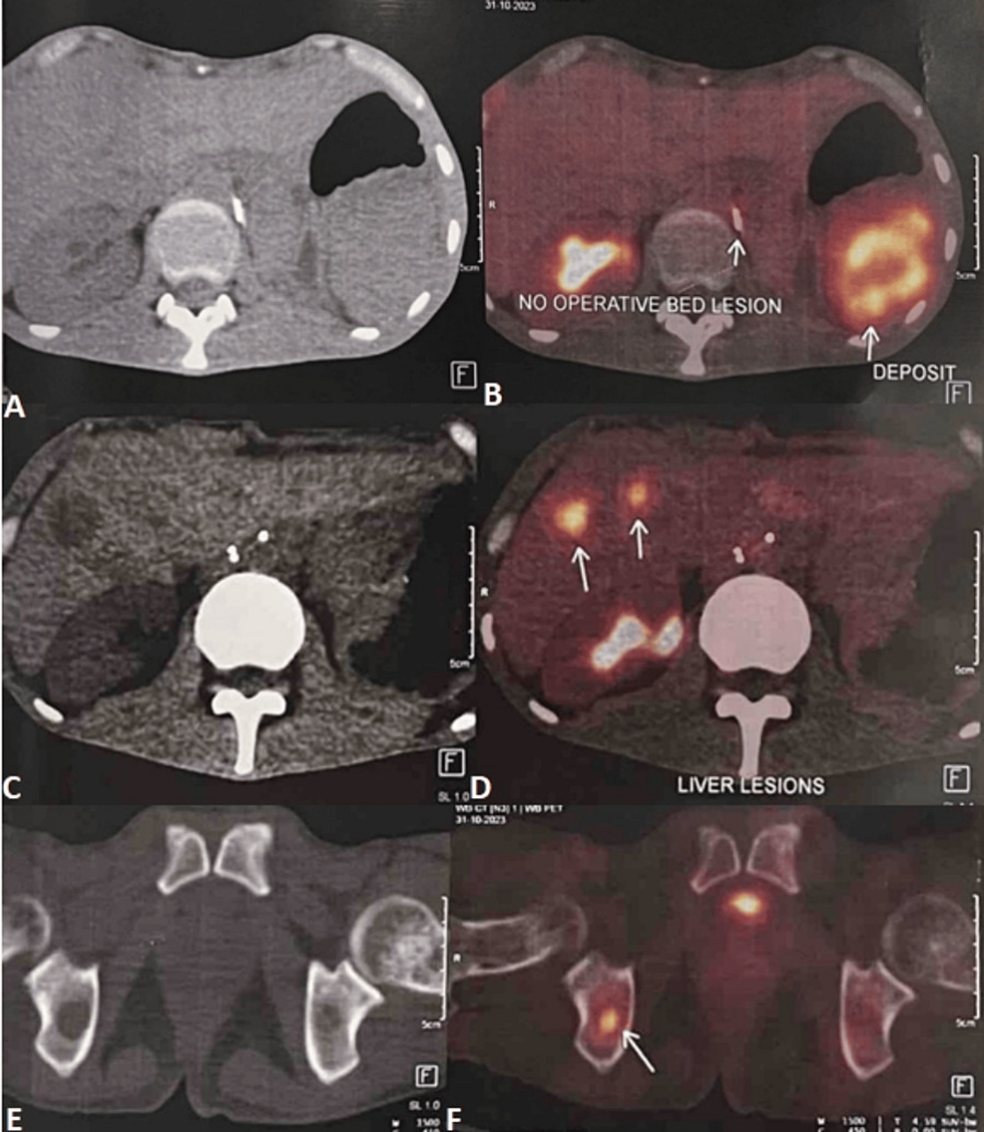
An 18F-FDG whole-body PET/CT scan reveals no lesions in the operative bed but shows FDG-avid hypodense perisplenic deposits (A, B, arrow), FDG-avid hypodense metastatic deposits in the liver (C, D, arrow), and an FDG-avid lytic lesion in the right ischium (E, F, arrow). F18-FDG: fluorine-18 fluorodeoxyglucose; PET: positron emission tomography

Radical nephrectomy with IVC thrombectomy under general and epidural anesthesia was done. A midline laparotomy incision extending from the xiphisternum to the pubic symphysis was made and extended bilaterally in the TR-horizontal plane. A large mass of approximate size 20x15 cm (CC x TR dimension) was identified in the left hypochondrium and lumbar region, not crossing the midline, adherent to the loop of the descending colon, and inferior mesenteric vein. The descending colon and liver were mobilized and the left renal artery was ligated. IVC was incised vertically and a thrombectomy was done. Adhered bowel segments were resected and colo-colic anastomosis was done. Homeostasis was achieved. A gross pathological examination of the surgical specimen revealed a well-circumscribed tumor in the mid and lower pole of the left kidney involving the cortex, medulla, renal sinus, and pelvicalyceal system, with tumor thrombus in the left renal vein and invasion of the perinephric adipose tissue and Gerota’s fascia. HPE showed a highly cellular neoplasm composed of fascicles of small ovoid cells with irregular nuclear contours and a scant amount of cytoplasm, hyperchromatic nuclei with irregular contours, and coarse vesicular chromatin (Figure 4A). Small foci of tumor cell necrosis and hemorrhage were also seen. Tumor infiltrates into the renal pelvis, perirenal fat, Gerota’s fascia, and left renal vein tumor thrombosis were also confirmed on HPE. Periodic acid-Schiff (PAS) stain highlighted the intracellular glycogen deposits. Immunohistochemistry (IHC) markers cluster of differentiation-99 (CD 99), synaptophysin (Figure 4B), B-cell lymphoma 2 (Bcl-2), and CD 56 were positive, and epithelial membrane antigen (EMA), pan-cytokeratin (pan-CK), transducin-like enhancer of split 1 (TLE1), CD 34, desmin, and S100 were negative. The final diagnosis of Ewing’s sarcoma of the left kidney was made based on HPE and IHC findings. Positron emission tomography (PET) CT performed one month after surgery revealed metastatic disease spread with fluorine-18 fluorodeoxyglucose (18F-FDG) avid hypodense lesions in both lobes of the liver, peri-splenic deposits, and FDG avid lytic bone lesions (Figures 5A-5F). A few small metastatic nodules were also seen in the bilateral lungs. The patient was started on chemotherapy comprising vincristine, cyclophosphamide, and doxorubicin.

**Figure 6:**
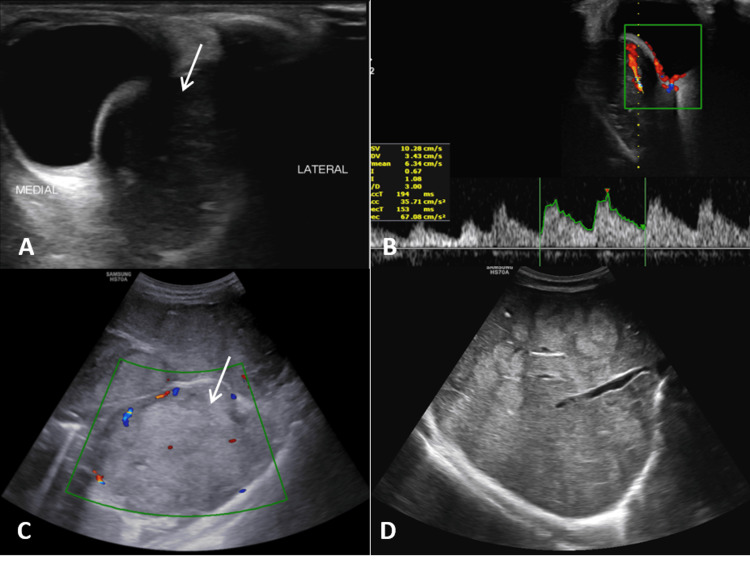
Ultrasound scan images of the left orbit (A, B) reveal a heterogeneously hypoechoic retro-bulbar mass lesion (arrow) showing vascularity on color Doppler with a low resistance waveform on spectral Doppler. Ultrasound of the abdomen and pelvis (C, D) shows a large heterogeneously hyperechoic lesion in the left renal fossa, abutting the spleen supero-laterally and the left lobe of the liver medially (C, arrow). Additionally, multiple round to oval hyperechoic focal lesions diffusely involving the liver parenchyma, most with peripheral halos, are observed (D). These findings are consistent with retro-bulbar, hepatic, and left renal fossa metastases.

**Figure 7:**
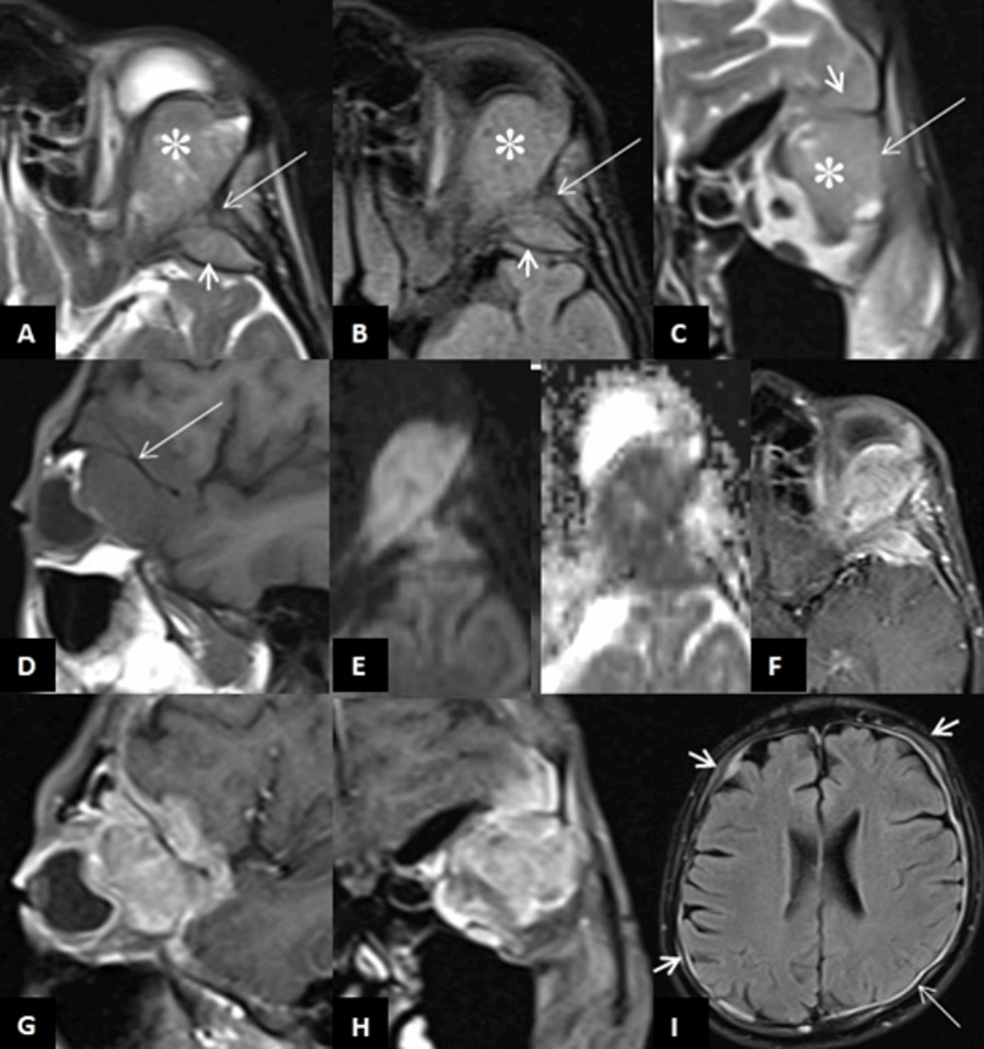
MRI images of the left orbital region (A-E) show a well-defined soft tissue lesion in the extra-conal compartment of the supero-lateral aspect of the left orbit. The lesion appears hyperintense on T2-weighted (A, C, asterisk) and FLAIR (B, asterisk) images, isointense to gray matter on T1-weighted images (D), and demonstrates diffusion restriction (E). There is an abnormal signal with erosions of the adjacent lateral wall of the left orbit, the lesser wing of the sphenoid, and the spheno-temporal buttress (A-D, long arrow), along with associated extra-dural soft tissue (A-C, short arrow). Heterogeneous enhancement of the orbital and extra-dural soft tissue lesions, as well as the involved bones, is noted on T1 fat-saturated images (F-H). Post-contrast fat-saturated axial FLAIR image (I) at the level of the body of the lateral ventricles also shows multiple abnormal calvarial lesions with adjacent soft tissue components (small arrows) and enhancing pachymeningeal thickening (long arrow). These findings are consistent with multiple skeletal metastases and a left extra-conal orbital soft tissue mass. FLAIR: fluid-attenuated inversion recovery

On subsequent follow-up two months after the surgery, the patient developed left temporal region pain, headache, and diminished vision in the left eye. Ultrasonography (USG) of the left eye revealed a well-defined hypoechoic soft tissue lesion of size 27x20 mm with vascularity in the retrobulbar region involving the lateral rectus muscle, compressing the eye globe antero-inferiorly and abutting the left optic nerve (Figures 6A-6B). USG abdomen revealed a large mass lesion of size approximately 9.2x8.8x7.2 cm (CCxAPxTR) in the left renal fossa near the spleen (Figures 6C-6D) suggestive of metastatic deposit with multiple hepatic metastases (Figure 6D). CE-MRI of the brain revealed an extra-conal soft tissue lesion with associated extra-dural soft tissue component and destructive changes in the adjacent lateral wall of the left orbit and lesser wing of the sphenoid (Figures 7A-7D). Multiple other bony lesions with diffuse dural thickening and enhancement were also seen (Figure 7I). These findings were consistent metastatic spread of the disease. The patient went into septic shock and succumbed to death the following month.

## Discussion

ESFT is made up of several tumors, the bulk of which are ESB, and also include tumors at extra-skeletal locations such as PNET, EES, and Askin tumors [2]. EES is extremely rare with an incidence of 0.4 per million [10]. ESFT often appears as a big, fast-expanding single mass and can occur anywhere in the body, affecting superficial or deep bone or soft tissues [11]. The kidney is the most frequently affected organ in the EES in the abdominal viscera [12]. As evidenced by our case [13], Ewing's sarcoma of the kidney is an exceedingly uncommon and aggressive neoplasm characterized by a rapid increase in lesion size and metastasis. Ewing's sarcoma of the kidney is classified as a subtype of small round cell tumors of the kidney, which also include synovial sarcoma, desmoplastic small round cell tumor, EES/PNET, lymphoma, clear cell sarcoma, carcinoid, monophasic Wilm's tumor, and synovial sarcoma [13]. EES exhibits a predilection for younger individuals and males [14]. Sarcomas affecting the kidney typically progresses from asymptomatic to symptomatic status en route to manifestation, and the mean size at the moment of diagnosis is between 5.5 and 23 cm [15,16]. The maximum dimension of the mass in our case was recorded as 20 cm intra-operatively. Clinically, flank pain is the most frequent initial manifestation, followed by hematuria and a palpable retroperitoneal (renal) which was also present in the index case [17]. Early metastatic disease and local recurrences are associated with a poor prognosis [14,18,15]. A majority of the EES cases present as large, dense solid mass soft tissue masses with ambiguous features in imaging studies [19]. Multiple studies have shown that these tumors show marked neovascularity, with the longest dimension exceeding 10 cm. These tumors usually infiltrate into the renal pelvis and also cause arterial and venous thrombosis [14,20]. They often do not cross the midline [2] but usually spread to the adjacent visceral fat with lymphovascular spread and metastatic lung and bone involvement, as seen in RCC [21]. In our case, the tumors had infiltrated the renal pelvis, perirenal fat, and Gerota’s fascia, with left renal vein and IVC tumor thrombosis.

USG is a readily available first line of investigation that shows renal EES as a heterogeneous mass of low echogenicity with internal vascularity on the Doppler study [22]. CECT is useful for identifying, characterizing, and staging renal EES, which is seen as a large, well-demarcated mass that is isodense to muscle showing heterogeneous post-contrast enhancement with necrotic hypodense areas [13]. The lesions may show few hyperdense areas in case of recent hemorrhage and faint amorphous type of calcifications occasionally [11,13]. MRI is the radiological modality of choice to assess EES tumors, including Ewing's sarcoma kidney [11]. EES typically shows high signal intensity on long repetition time (TR) images with high signal intensity areas indicating necrosis or cystic changes and appear isointense to hypointense on T1-weighted images with heterogeneous post-contrast enhancement [23,24]. The lesion also shows intermediate signal intensity areas on long TR images, indicating high cellularity, often with significant enhancement due to tumor hypervascularity [14]. Susceptibility-weighted sequences often reveal areas of hemorrhages. Although we may observe features like fluid-fluid levels, pseudo-capsules, and serpentine high-flow vascular channels, they are not consistently sensitive or specific for EES [19]. ^18^F-FDG PET-CT is crucial for staging and detection of nodal and distant metastatic disease [25,26] with a reported sensitivity of 87%, specific of 97%, and accuracy of 94% for detection of distant metastasis in ES by Gerth et al. [26]. FDG PET-CT also plays an important role in the assessment of the treatment response and recurrent disease in patients undergoing chemotherapy. PET-CT, in our case, was performed one month after the surgery and showed a clear operative bed with peri-splenic metastatic deposit and lung, liver, and skeletal metastases. Usually, renal EES can be seen as large renal mass lesions with uneven post-contrast enhancement, showing areas of necrosis and bleeding, and irregular septations with renal vein or IVC thrombosis [27].

Clinically, EES is more likely than RCC in the presence of a highly aggressive tumor with early metastasis occurring in the second and third decades. Lung and bone are the most frequent metastases, occurring in as many as 80% and 40% of these patients, respectively [13,24,28,29]. Lymph nodes are an uncommon site for metastases. At the time of diagnosis, one-third of patients exhibit tumor thrombi in the renal vein or IVC [30]. There was left renal vein and IVC thrombosis in our patient at the time of diagnosis, with few suspicious bone metastases. The patient rapidly developed metastatic spread to the liver, peri-splenic region, and multiple bones in the follow-up just after one month of the surgery.

Differential diagnosis on imaging for a solitary, aggressive, primary renal tumor comprises RCC, transitional cell carcinoma, lymphoma, and mesenchymal malignancies like osteosarcomas, synovial sarcoma, and leiomyosarcoma [17]. Necrosis, hemorrhage, and calcification giving rise to a heterogeneous appearance are seen in both RCC and sarcomas. Areas of calcification or zones of ossification are seen in osteosarcomas [17]. Renal shape is maintained in transitional cell carcinoma [31]. Homogeneous enhancement is often seen in lymphoma [32].

Histological examination of renal EES reveals small round to oval blue cells with large hyperchromic nuclei and minimal cytoplasm. IHC with a spectrum of markers is used in the diagnosis of EES, including highly sensitive but non-specific CD99 antigen, and more useful neural markers such as vimentin, neuron-specific enolase (NSE), S-100 protein, and synaptophysin [22,33]. Molecular genetic tests in EES show Ewing’s sarcoma-specific reciprocal translocation t(11;22)(q24;q12) present in 90% of cases with formation of the EWS-ETS fusion gene [2,34,35]. This creates a fusion between the FLI gene on 11q24 and the EWSR1 gene on 22q12 with the formation of a more specific DNA-binding transcription factor EWS/FLI-1 [2,34]. HPE in the present case showed highly cellular neoplasm composed of fascicles of small ovoid cells with hyperchromatic irregular nuclei with scanty cytoplasm nuclei and coarse vesicular chromatin. IHC markers CD99 and synaptophysin were positive favoring diagnosis of EES. Due to its unavailability, molecular genetic studies were not done in our patient.

Surgical resection and adjuvant chemotherapy remain the mainstay of the treatment [30]. The standard chemotherapy protocol in patients with Ewing’s sarcoma comprises four drugs: doxorubicin (75 mg per square meter), cyclophosphamide (1200 mg per square meter), vincristine (2 mg per square meter of body surface area), and dactinomycin (1.25 mg per square meter) [36]. Experimental therapy with the addition of ifosfamide (1800 mg per square meter per day for five days) and etoposide (100 mg per square meter per day for five days) to standard protocol has shown substantial improvement in patients with non-metastatic Ewing’s sarcoma, especially in large primary tumors [36]. Molecular targeted therapy has shown significant promise with the use of insulin-like growth factor 1 receptor antibody [13]. Postoperative radiotherapy is advised only when surgical margins are not sufficient. This tumor exhibits a dismal prognosis with a median survival of only 15 months [30].

## Conclusions

Ewing’s sarcoma of the kidney is a rare and highly aggressive neoplasm that predominantly affects young adults and children. Due to its rarity and non-specific presentation, diagnosis remains a taxing task. Radiological and nuclear imaging, HPE, and molecular genetics are necessary for an accurate and timely diagnosis. Management involves a multidisciplinary approach that includes surgery, chemotherapy, and sometimes radiotherapy. The prognosis remains guarded due to the high potential for metastasis and recurrence. Ongoing research into the molecular mechanisms of Ewing’s sarcoma and the development of targeted therapies holds promise for improving outcomes. Early detection and tailored therapeutic strategies are essential to enhancing survival rates and quality of life for patients.
